# Cerebellar network organization across the human menstrual cycle

**DOI:** 10.1038/s41598-020-77779-4

**Published:** 2020-11-26

**Authors:** Morgan Fitzgerald, Laura Pritschet, Tyler Santander, Scott T. Grafton, Emily G. Jacobs

**Affiliations:** 1 Department of Psychological and Brain Sciences, University of California, Santa Barbara, Santa Barbara, CA 93106 USA; 2 Institute for Collaborative Biotechnologies, University of California, Santa Barbara, USA; 3 Neuroscience Research Institute, University of California, Santa Barbara, USA

**Keywords:** Neuroscience, Endocrinology

## Abstract

The cerebellum contains the vast majority of neurons in the brain and houses distinct functional networks that constitute at least two homotopic maps of cerebral networks. It is also a major site of sex steroid hormone action. While the functional organization of the human cerebellum has been characterized, the influence of sex steroid hormones on intrinsic cerebellar network dynamics has yet to be established. Here we investigated the extent to which endogenous fluctuations in estradiol and progesterone alter functional cerebellar networks at rest in a woman densely sampled over a complete menstrual cycle (30 consecutive days). Edgewise regression analysis revealed robust negative associations between progesterone and cerebellar coherence. Graph theory metrics probed sex hormones’ influence on topological brain states, revealing relationships between sex hormones and within-network integration in Ventral Attention, Dorsal Attention, and SomatoMotor Networks. Together these results suggest that the intrinsic dynamics of the cerebellum are intimately tied to day-by-day changes in sex hormones.

## Introduction

Although its Latin name means “little brain”, the human cerebellum contains nearly four times as many neurons as the cerebral cortex, with the posterior and lateral regions greatly expanded in humans relative to apes^1^. Engagement of the cerebellum during cognitive control tasks challenges the classic notion that the cerebellum is solely involved in motor coordination,rather, it appears to coordinate a broad range of higher-order cognitive functions^2–5^. Multiple closed-loop circuits between the cerebellum and cortex, including non-motor regions of the prefrontal cortex (PFC^6,7^, provide an anatomical basis for cerebellar involvement in cognition including learning, memory, and decision making^8–10^. Thus, the tradition of branding the cerebellum as a purely motor-associated region is becoming increasingly obsolete.

Allen et al.^11^ demonstrated the utility of using functional magnetic resonance imaging (fMRI) to assess functional synchrony between the cerebellum and the cerebral cortex, finding that low-frequency signal fluctuations in the cerebellum correlate with signal fluctuations in subcortical, parietal, and frontal regions. Topographically distinct fronto-cerebellar circuits involving the dorsolateral PFC, medial PFC, and anterior PFC have since been identified^12^. A seminal fMRI study by Buckner et al.^13^ revealed that the cerebellum houses at least two complete homotopic maps of cortical networks. Specifically, the cerebellum contains hubs of major functional brain networks including the Default Mode Network (DMN), Frontal Control Network (FCN), SomatoMotor Network (SMN), Dorsal Attention Network (DAN), Ventral Attention Network (VAN), and Limbic Network^13^.

Accumulating evidence implicates the cerebellum as a major site of sex steroid hormone action. The developing cerebellum exhibits de novo synthesis of estrogen and progesterone^14–16^, and estrogen influences the formation of dendritic spines and synapses through regulation of microglia^17^. The adult cerebellum demonstrates a rich expression of estrogen receptors (ER and progesterone receptors (PR^18–21^) Evidence suggests that sex hormones not only influence the formation of cerebellar neuronal circuitry during neonatal development, but also modulate cerebellar functioning later in life. The vast majority of Purkinje cells, the major output units of the cerebellum, densely express ERβ^20,21^, and estradiol has been shown to improve cerebellar memory formation by enhancing long-term potentiation and augmenting cerebellar synapse formation^22^. Granule cells, the most prevalent neuronal population in the cerebellum, are in direct connection with gamma-Aminobutyric acid(GABA)ergic Golgi cells^23^ and progesterone enhances GABA signaling^24,25^. Although much attention has been paid to sex hormones’ ability to regulate spinogenesis, synaptic plasticity, and neural activity in cortex^26–28^, sex hormones’ role in the cerebellum is now gaining increased recognition.

Despite preclinical evidence that sex hormones regulate cerebellar function, human studies are lacking. Across a typical human menstrual cycle, spanning 25–30 days, women experience an eightfold increase in estrogen and an 80-fold increase in progesterone. These in vivo changes in sex hormones have been shown to modulate brain structure, task-evoked cortical activity, and performance on cognitive tasks^29–31^. However, most menstrual cycle studies sparsely sample women at discrete timepoints (e.g. 2–4 times), obscuring the rhythmic changes in hormone production across a complete cycle^32,33^. The field of network neuroscience has begun to use dense-sampling methods to probe the properties of the human brain over unprecedented timescales to study the dynamic time-varying properties of the human brain over days, weeks, months, and years^34–38^. In a recent dense-sampling study from our group, a woman underwent 30 consecutive days of brain imaging and venipuncture across a complete menstrual cycle, revealing estradiol and progesterone’s ability to modulate widespread patterns of connectivity across the cortex^37,39^. Given the sensitivity of the cerebral cortex to endogenous fluctuations in sex steroid hormones^33,37,39–41^ and accumulating evidence for sex hormone action in the cerebellum^42,43^, here we tested the hypothesis that sex hormones impact the intrinsic day-to-day dynamics of cerebellar circuits.

In this dense-sampling, deep-phenotyping study, we examined whether day-by-day variation in sex hormones across a complete menstrual cycle modulates cerebellar functional connectivity and cerebellar network topologies. Results reveal that estradiol and progesterone are associated with daily variation in coherence across the cerebellum and both intra- and inter-network integration, providing insight into how sex hormones shape the intrinsic properties of the human cerebellum.

## Results

A healthy, naturally-cycling female (author L.P.; age 23) underwent venipuncture and MRI scanning for 30 consecutive days. The full dataset consists of daily mood, diet, and behavioral assessments; task-based and resting-state fMRI; structural MRI; and serum assessments of pituitary gonadotropins and ovarian sex hormones^37^. Neuroimaging data, daily behavioral assessments, and analysis code is publicly available (see “Data/code availability”).

### Endocrine assessments

**Figure 1:**
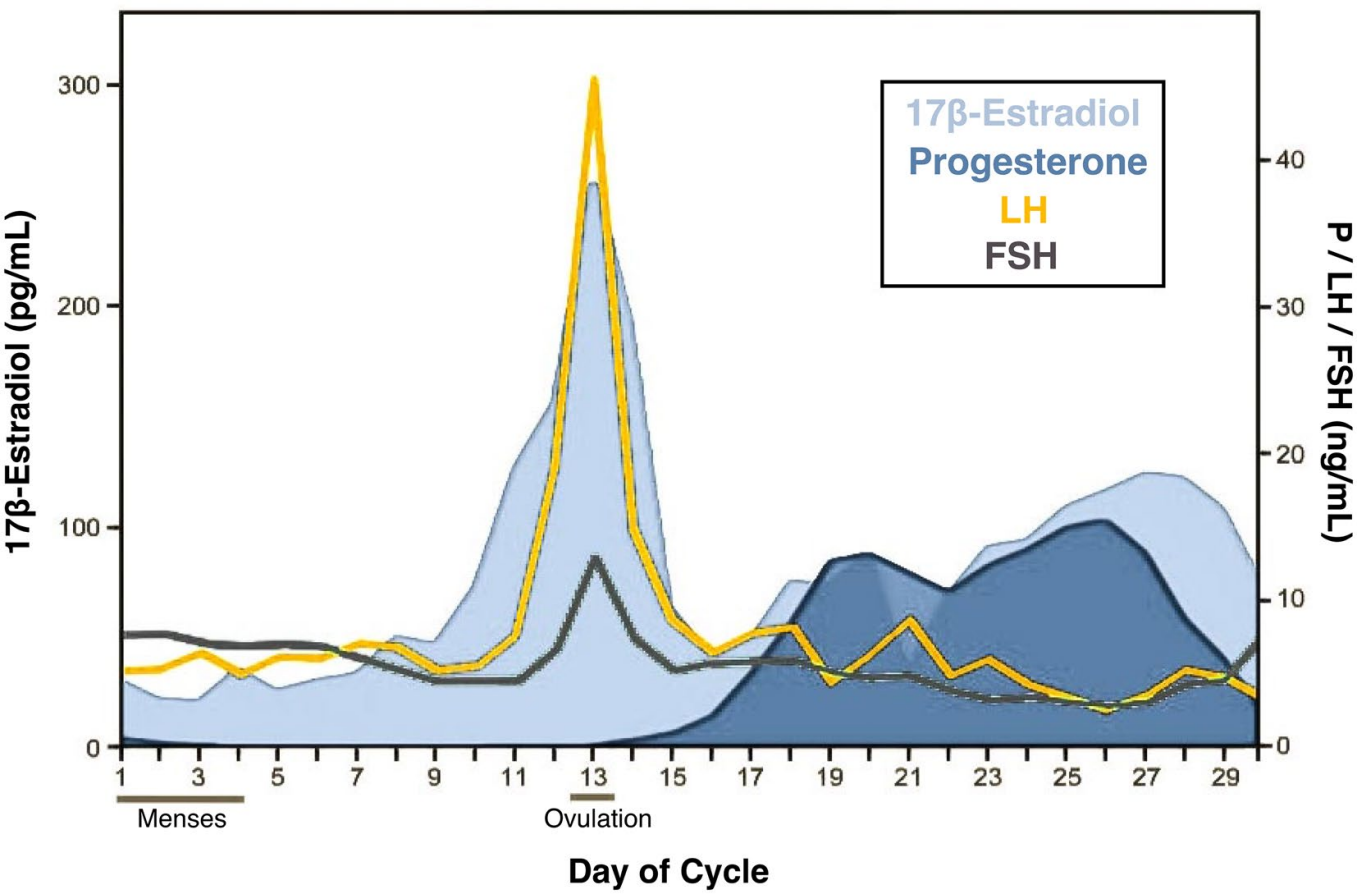
Participant’s hormone concentrations plotted by day of cycle. 17β-estradiol, progesterone, luteinizing hormone (LH), and follicle stimulating hormone (FSH) concentrations fell within standard ranges. (Adapted from^37^).

Analysis of daily sex hormone (by liquid-chromatography mass-spectrometry) and gonadotropin (by chemiluminescent immunoassay) concentrations confirmed the expected rhythmic changes of a typical menstrual cycle. All hormones fell within normal ranges (Table S1), with a total cycle length of 27 days. Serum levels of estradiol and progesterone were lowest during menses (day 1–4) and peaked in the late follicular (estradiol) and late luteal (progesterone) phases (Fig. 1). Progesterone concentrations surpassed 5 ng/mL in the luteal phase, signaling an ovulatory cycle^44^.

### Temporal dependencies between sex hormones and edgewise connectivity

To begin, we tested the hypothesis that cerebellar functional connectivity at rest is associated with intrinsic fluctuations in estradiol and progesterone in a day-by-day fashion. Given the pronounced expression of PR within the cerebellum and the ability of progesterone to augment inhibitory responses within cerebellar neurons^19,24,42^, we predicted decreases in cerebellar functional connectivity as progesterone concentrations increase across the cycle, in keeping with results previously found in the cerebrum^37^. Further, we predicted estradiol would augment cerebellar coherence. For each session, the cerebellum was parcellated into 99 nodes and then were spatially mapped to a seven-network atlas^13,45^. A summary time-course was extracted from each node, data were temporally filtered, and 99 × 99 functional association matrices were derived via magnitude-squared coherence (FDR-thresholded at *q* < 0.05; see “Methods and materials” for full description of preprocessing and connectivity estimation). Next, we specified edgewise regression models, regressing coherence against estradiol and progesterone over the 30-day study. Data were *Z*-scored prior to analysis and models were thresholded against empirical null distributions generated through 10,000 iterations of nonparametric permutation testing. Results reported below survived a conservative threshold of *p* < 0.001. For a visualization of day-by-day variation in network topologies across the 30-day experiment and further depiction of network-specific relationships between hormones and edgewise connectivity, see Supplementary Materials (Figures S1, S2).

**Figure 2:**
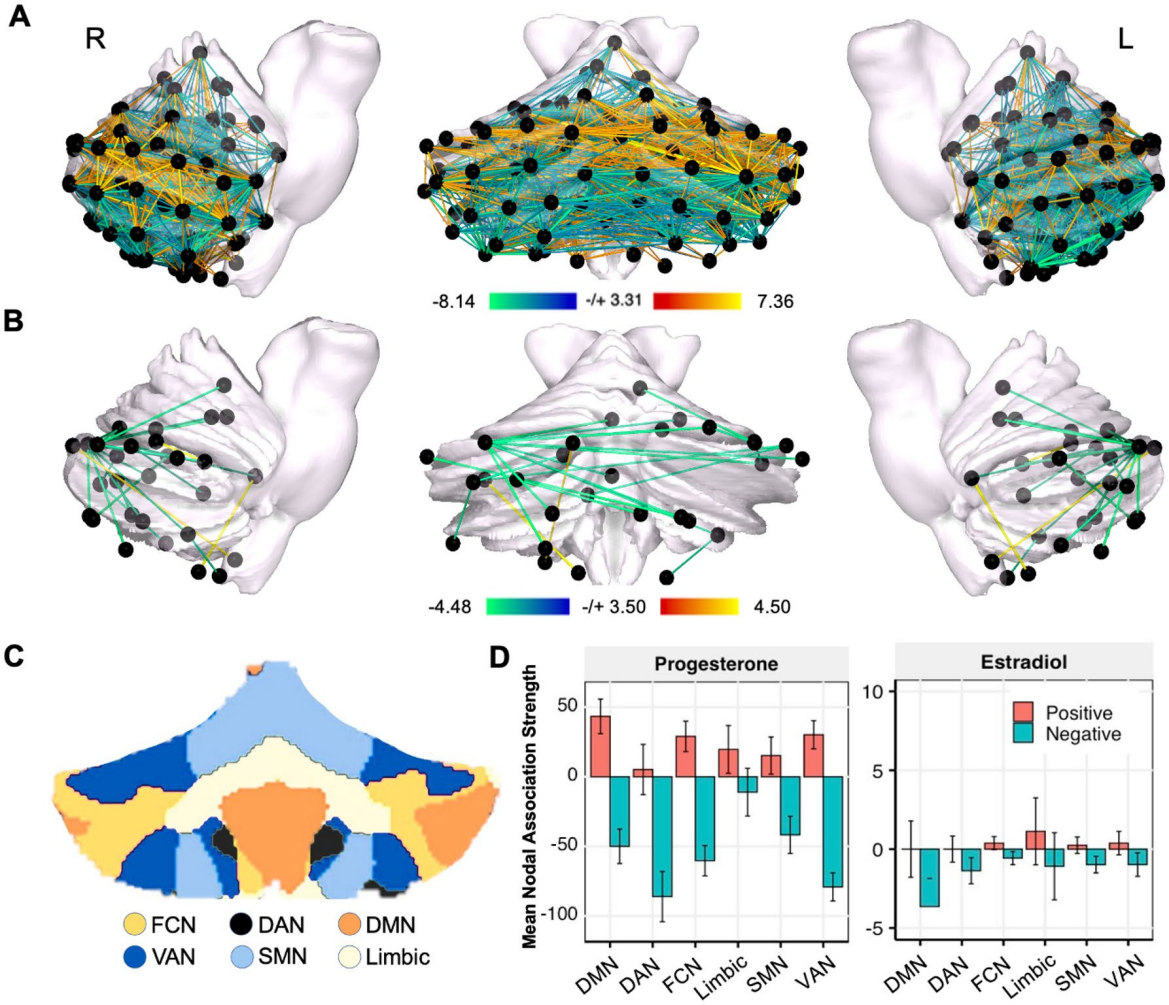
Whole cerebellum functional connectivity at rest is associated with intrinsic fluctuations in estradiol and progesterone. (**A**) Day-by-day associations between progesterone and coherence. Hotter colors indicate increased coherence with higher concentrations of progesterone; cool colors indicate the reverse. Results are empirically-thresholded via 10,000 iterations of nonparametric permutation testing (*p* < .001*).* Nodes without significant edges are omitted for clarity. Values presented represent beta divided by the standard error, representing relative effect sizes for the standardized regression (**B**) Day-by-day associations between estradiol and coherence. (**C**) Cerebellar parcellations were defined by Buckner et al. seven-network atlas^13^. Note that the Visual Network is not represented in the cerebellum. (**D**) Mean nodal association strengths by network and hormone, calculated by averaging edgewise connectivity for all nodes in a given network associated with progesterone or estradiol. Error bars give 95% confidence intervals. ‘Positive’ refers to the average magnitude of positive associations (e.g. stronger coherence with higher estradiol). Note progesterone had greater associations with edgewise connectivity as reflected in the y-axis range. Abbreviations: DMN, Default Mode Network; DAN, Dorsal Attention Network; FCN, Frontal Control Network; SMN, SomatoMotor Network; VAN, Ventral Attention Network. Statistical maps of edgewise coherence v. hormones were visualized using the Surf Ice software (https://www.nitrc.org/projects/surfice/).

In keeping with our predictions, progesterone yielded a widespread pattern of robust inverse associations across the cerebellum, such that whole-cerebellar coherence decreased as progesterone concentrations rose (Fig. 2A). Next, the average magnitude of brain-hormone association was summarized by network (using the Buckner seven-network parcellation; Fig. 2C). Although all networks demonstrate some degree of positive associations over time, the strength of negative associations was larger in magnitude and significantly nonzero across nearly all networks, as depicted by mean nodal association strengths (Fig. 2D). The DMN was unique in that the degree of negative and positive associations were nearly equal while the Limbic Network was the only network for which positive associations were greater than negative associations. Together, these results align with previous findings in the cerebrum indicating strong decreases in whole-brain functional connectivity as progesterone concentrations increase across the cycle^37^.

In contrast to our predictions, we observed sparse and predominantly negative associations between estradiol and cerebellar coherence (Fig. 2B). All cerebellar networks exhibited some degree of significantly negative associations with estradiol (95% CIs not intersecting zero), particularly the DMN and DAN (Fig. 2D). The Limbic Network was unique again in that it demonstrated a heterogenous response with positive and negative association strengths (Fig. 2D). These findings suggest that, within the cerebellum, increases in estradiol are associated with sparse decreases in connectivity, a pattern that differs from the positive associations observed across the cerebrum^37^.

### Temporal dependencies between sex hormones and network topology

Given the widespread associations between whole-cerebellar coherence and sex hormones, we examined *topological states* of cerebellar networks to capture the extent of brain-hormone interactions at the network level. Topological states were quantified using common graph theory metrics, including estimates of between-network integration (*participation*) and within-network integration (*global efficiency*). See Supplementary Materials (Tables S2 and S3) for a complete summary of all results.

**Figure 3:**
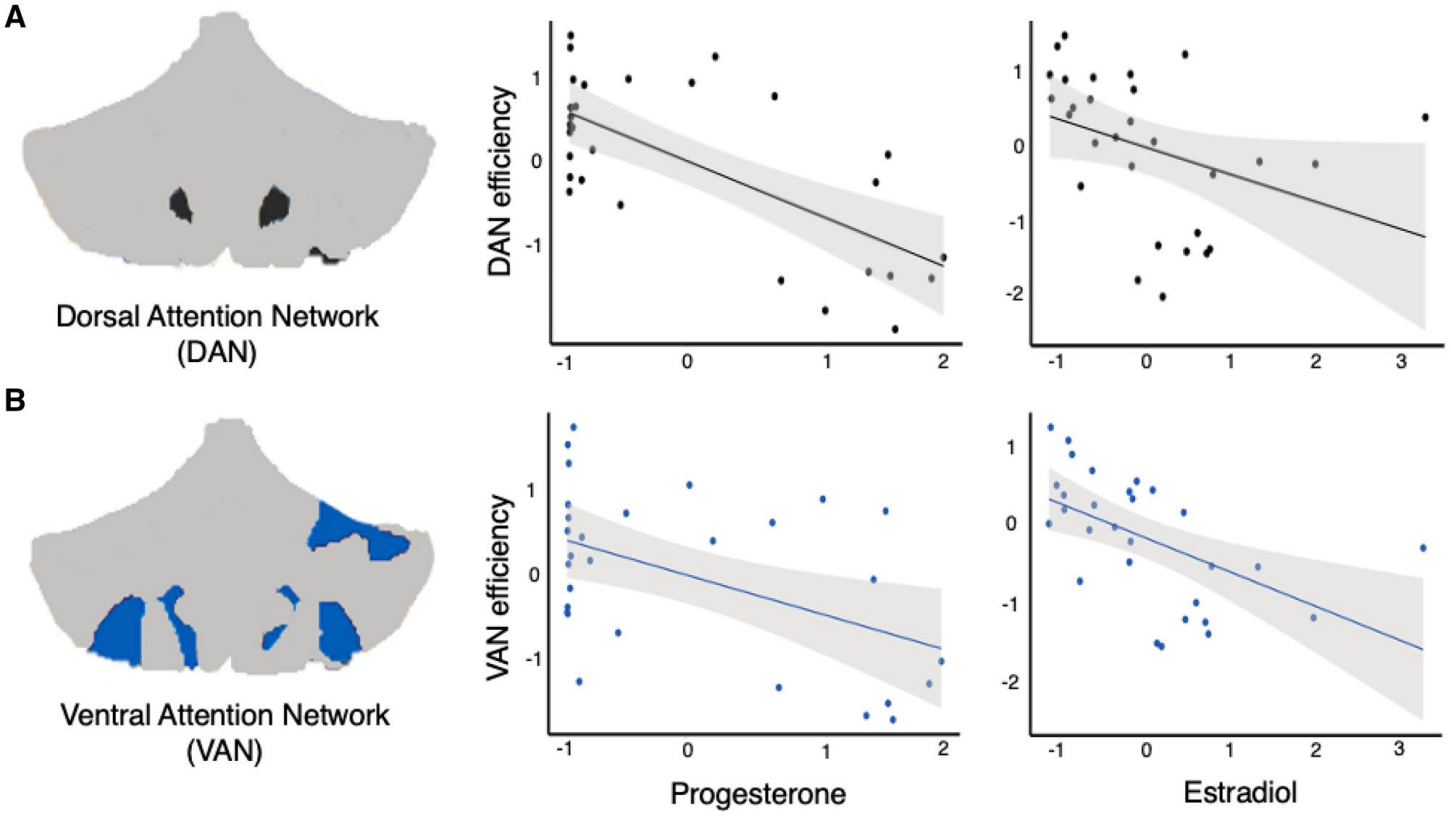
Graph theory metrics reveal relationships between sex hormones and intra-network integration. (**A**) Illustration (left) depicts nodes belonging to the Dorsal Attention Network (DAN). Global efficiency, a measure of within-network integration, was calculated to reflect the ostensible ease of information transfer across clusters inside a given network and was regressed against sex hormone concentrations. Here, scatter plots depict significant associations (p < .05) between progesterone (middle) or estradiol (right) with DAN efficiency. (**B**) Illustration (left) depicts nodes belonging to the Ventral Attention Network (VAN). Here, scatter plots depict significant associations (p < .05) between progesterone (middle) or estradiol (right) with VAN efficiency. Note that data are z-scored before models are fit. For a complete description of results, see Figure S3 and Tables S2, S3. Cerebellar parcellations depicted in A and B were defined from the Buckner et al. seven-network atlas (2011) and scatter plots were generated in R (version 3.4.4).

To investigate day-by-day relationships between topological states of each network and hormone fluctuations across the menstrual cycle, a series of linear regression analyses were conducted. After initially fitting linear models to the dataset, an inspection of residual densities revealed experiment day one as a frequently poor fit (median absolute deviation > 3); it was therefore removed from the analyses reported here. Remaining data were *Z*-scored, and network metrics were residualized on motion (mean FWD) prior to model estimation (*p*-values reported are FDR-corrected at the level of *q* < 0.05). Regression models revealed that progesterone was associated with DAN ($\beta = -.48, \;SE= .15, \;t= -3.29, \;p= .008;$ Fig. 3A) and VAN efficiency ($\beta = -.39, \;SE= .17, \; t= -2.32, \; p= .028$; Fig. 3B), and both model fits were significant (DAN: F(1, 28) = 10.82, $p= .008, \; { R}_{Adjusted}^{2}= .25;$ VAN: F(1, 28) = 5.38, $p= .028, \; { R}_{Adjusted}^{2}= .13)$. Between-network integration (as measured by participation) for FCN was also associated with progesterone ($\beta = -.43, \; SE= .16, \; t= -2.70, \; p= .026$; Figure S3B), and the model fit was significant (F(1, 28) = 7.28, $p= .026, \; { R}_{Adjusted}^{2}= .18)$. In sum, dynamic changes in progesterone across the menstrual cycle were associated with intra- and inter-network integration of functional brain networks.

Estradiol was associated with global efficiency within DAN ($$\beta = -.37, \; SE= .16, \; t= -2.36, \; p=.028;$$ Fig. 3A), VAN($$\beta = -.54, \; SE= .16, \; t= -3.51, \; p= .008;$$ Fig. 3B) $$\text{ and SMN }(\beta = -.39, \; SE= .17, \; t= -2.33, \; p=.028;$$ Figure S3A). This suggests that the within-network integration (as measured by global efficiency) of major functional brain networks is negatively associated with estradiol across the cycle. Overall model fits were significant for the DAN $$(\text{F}(1, 28)=5.55, \; p= .028$$, $${R}_{Adjusted}^{2}= .14),$$ VAN $$(\text{F}(1, 28)=12.33, \; p=$$ .008, $${R}_{Adjusted}^{2}= .28)$$ and SMN $$\left(\text{F}\left(1, 28\right)= 5.43, \; p = .028, \; {R}_{Adjusted}^{2}= .13\right).$$ Model fits for the remaining networks were poor and did not demonstrate significant associations with estradiol (Tables S2, S3). These data are in agreement with our edgewise regression analysis depicting decreased whole-cerebellar coherence with increasing estradiol.

## Discussion

In this dense-sampling, deep-phenotyping project, a naturally-cycling female underwent resting-state fMRI and venipuncture for 30 consecutive days, capturing the dynamic endocrine changes that unfold over the course of a complete menstrual cycle. Edgewise regression analyses illustrate robust negative associations between progesterone and cerebellar coherence, and to a lesser degree, sparse negative associations between estradiol and cerebellar coherence. Graph theory metrics were used to examine cerebellar network topologies, indicating negative associations between estradiol and global efficiency within the DAN, VAN, and SMN, between progesterone and global efficiency within the DAN and VAN, and between progesterone and participation of the FCN. Together, these results reveal that estradiol and progesterone are associated with cerebellar functional connectivity and network topology, providing insight into the relationship between sex hormones and the intrinsic dynamics of the human cerebellum.

Sex steroid hormones influence *cortical* functional connectivity and network topography, as demonstrated by parallel analysis across the cerebrum^37^. While our predictions for the impact of gonadal hormones on cerebellar network dynamics were analogous to the effect of sex hormones on cerebral networks, our data suggests that day-by-day associations of hormones and cerebellar functional connectivity at rest diverge somewhat from that of the cerebrum. Progesterone’s effects demonstrated robust negative associations with coherence in both the cerebellum and cortex^37^, but the association strengths were considerably higher in the cerebellum (tenfold minimum), hinting that progesterone may have a greater influence on cerebellar coherence. In the cortex, estradiol concentrations were associated with increased connectivity across all major networks, particularly the DMN and DAN^37^, but this effect was absent in the cerebellum where estradiol was associated with *reductions* in connectivity across networks. This implies that estradiol might enhance functional connectivity within the cortex while simultaneously decreasing connectivity within the cerebellum. Critically, the mechanisms driving the unique effects of these hormones in the cerebellum have yet to be characterized.

The human cerebellum shows rich expression of PR^19^ and higher progesterone concentrations than the cingulate cortex^46^. This could account for the amplified effects of progesterone on cerebellar network coherence relative to cortex. On a molecular level, progesterone potentiates GABAergic activity^25^ and enhances the response of Purkinje cells to GABA transmission^24,47^, which could explain the robust decrease in cerebellar functional connectivity as progesterone concentrations increased across the cycle. In addition to Purkinje cells, the cerebellum houses GABAergic interneurons and GABAergic Golgi cells. Interneurons play a key role in cerebellum functioning and govern Purkinje cell output^48–50^, while Golgi cells synapse directly onto granule cells, the most numerous neurons in the cerebellum^23^. Thus, progesterone-driven enhancement of GABA signaling could enhance cerebellar inhibition across the cycle by selectively enhancing the activity of either interneurons or Golgi cells. GABAergic activation by progesterone also counters estradiol-induced increases in neuronal excitability^51^, providing an additional potential mechanism for the observed progesterone-associated decreases in functional coherence.

Our results suggest that estradiol has opposing effects across cerebellar and cortical networks. Although this result is in contrast to our predictions, we present two possible explanations. First, the disparity in estradiol-coherence associations may be attributable to striking differences in ER subtype distributions between the cerebrum and cerebellum. Cerebellar Purkinje cells only express ERβ^20,21,52^ in contrast to the cerebrum which exhibits robust expression of ERα and ERβ^20,21^. ERα and ERβ have similar binding affinities for estradiol^53,54^, but the two receptor subtypes diverge in their physiological roles and interactions^54–59^. Though speculative the divergent effects of estradiol seen across the cortex and cerebellum could in part be attributable to the cerebellum’s unique receptor profile. Second, estradiol’s divergent effects could also be mediated through the hormone’s action on non-neuronal cell populations, as the cortex and cerebellum house divergent populations of microglia and astrocytes. Microglia demonstrate organizational and morphological differences across brain localities, with cerebellar microglia being sparsely distributed and having less branched morphology relative to cortical populations^60^. Astrocyte populations also show regional differences, with cerebellar astrocytes being outnumbered by neurons, while cortical astrocytes greatly outnumber neurons^61^. Estrogen modulates the formation of dendritic spines and synapses through regulation of microglia^17^, and stimulates calcium release and progesterone synthesis in astrocytes^62^, presenting additional mechanisms to explain the observed discrepancy in estradiol-coherence associations. Additional research is needed to definitively link differences in receptor expression or cellular populations to variability observed at the mesoscopic level of functional networks.

Sex hormone’s modulation of cerebellar functional connectivity has implications for understanding human brain organization across the lifespan. The cerebellum is involved in a broad spectrum of cognitive functions including learning, memory, and decision making^8–10^. Further, some age-related declines in cognitive function may be attributable to neuronal changes in the cerebellum. Cerebellar volume declines progressively with advanced age^63–67^ and these age-associated volumetric changes may precede those found in subcortical structures such as the hippocampus^68^. Sex differences in age-related declines of cerebellar volume have been observed, where midlife women approaching menopause show reductions in cerebellar lobe and vermis volumes relative to age-matched men^69,70^, hinting at a potential role of sex steroid hormones in cerebellar aging. While our results establish a relationship between sex hormones and cerebellar functional brain network organization, future studies should investigate how hormone-mediated changes influence cognition and whether morphological changes in the human cerebellum occur across the menstrual cycle or other major hormone transition states (e.g. menopause).

Sex hormone action in the cerebellum is also implicated in neurodegenerative diseases. Alzheimer’s Disease (AD) is a progressive neurodegenerative disease that exhibits profound sex-differences, with two thirds of sufferers being women^71^. Wegiel et al.^72^ identified significant reductions in cerebellar volume as a feature of AD pathology. In the progression to AD, the cerebellum undergoes significant morphological alterations, including extensive loss of Purkinje cells, reductions in dendritic spines, and altered dendritic arborization^73,74^. Notably, sex hormone receptor expression (ER and PR) within the cerebellum is highly localized to Purkinje cells^20,75^. Future studies should investigate whether sex hormones play a role AD-related cerebellar atrophy. Given sex hormones’ ability to shape cerebellar dynamics in a healthy brain, they might also play a role in age- and disease-related cerebellar degeneration.

Limitations of the current study should be considered when interpreting these findings and outlining future investigations. First, the cerebellum is a challenging structure to probe due to its low signal-to-noise ratio and the fact that it contains the vast majority of neurons in only one-ninth of the volume of the cortex^76,77^. These challenges result in the cerebellum requiring twice as much resting-state data to achieve the same level of reliability as the cerebrum^76^. Here, a daily 10-min resting-state scan was collected from a single individual for 30 consecutive days, providing a longitudinal dataset to examine cerebellar functional connectivity. As the amount of data collection needed to achieve intra/inter-reliability is debated within the field^78^, future work should explore how robust these results are to varying scanning durations.

Second, while the majority of previous cerebellum work has relied on anatomical parcellations, we chose a function-based parcellation to capture the cerebellum’s functional subdivisions. The parcellation we applied outperformed the standard voxel-based approach and other existing cerebellar atlases across measures of node homogeneity, accuracy of functional connectivity representation, and individual identification. However, the parcellation was more accurate when identifying cerebro-cerebellar functional connectivity relative to cerebellar connectivity^45^, suggesting room for improvement when assessing *cerebellar* coherence. Additionally, a recent publication by Seitzman et al.^79^ proposes that applying a novel ‘winner-takes-all’ partitioning method within the cerebellum produces functionally constrained nodes at an unmatched degree of validity across multiple data sets and anatomical atlases. Our results are reported with respect to one parcellation^45^, therefore, future work should consider applying multiple parcellations to individual datasets to determine whether robust validity of cerebellar connectivity can be obtained. Additionally, group-based fixed atlases may lead to loss in individual-level specificity, unable to capture potentially meaningful changes in the parcellations themselves^80^. Future experiments would benefit from deriving cerebellar functional networks in an individualized manner.

Third, our preprocessing pipeline used a spatial smoothing filter (4 mm Gaussian kernel) in effort to achieve a higher signal-to-noise ratio, but application of the smoothing kernel could partially obscure spatial specificity^81–83^. Note that we repeated our edgewise regression analyses without a smoothing kernel and results largely paralleled findings reported here (see Supplementary Materials Figure S4).

Fourth, resting-state scans are highly sensitive to motion. However, motion was limited to fewer than 130 microns per-day on average and robust nuisance signal regression procedures were implemented to reduce motion bias. We also took steps to remove day-by-day motion tendencies from our measures of network topology prior to analysis with hormones. However, replication studies using additional motion correction strategies, such as removal of physiological noise contaminants, would further strengthen these results^84^. Note that day-by-day motion was quantified using a recent filtering approach aimed at reducing high-frequency contamination from motion estimates^84^,this approach confirmed consistently low motion throughout the experiment (Figure S5).

Fifth, analyses reported here model the relationship of two major ovarian hormones (i.e. estradiol and progesterone) independently, and thus are unable to address how changes in the *ratio* of the two steroids impact cerebellar network dynamics. Future work should consider the association between network dynamics and hormone ratios.

Finally, this study densely sampled a single individual over one menstrual cycle, which hinders the generalizability of these findings to a larger population. Follow-up studies that use sparse-sampling methods to investigate cerebellar dynamics in larger samples of women and men across different hormone states (e.g. menstrual cycle, oral contraceptive use, menopause, andropause) will strengthen our understanding of sex steroid hormone action in cerebellar function.

## Conclusion

Over the past 30 years, cognitive neuroscience has established the cerebellum’s integral role in cognition^85,86^, dissolving the notion that it is a purely motor-associated region. A parallel literature suggests that the cerebellum is a prominent target of sex hormones^42,43^. Here, we demonstrate that endogenous fluctuations in estrogen and progesterone over the menstrual cycle impact the intrinsic network properties of the cerebellum. Thus, it is critical to consider the endogenous hormone milieu when investigating functional network properties of the human brain.

## Methods and materials

### Participant

A right-handed Caucasian female, aged 23 years, underwent venipuncture and magnetic resonance imaging (MRI) scans for 30 consecutive days. The participant had no history of neuropsychiatric diagnosis, endocrine disorders, or prior head trauma. She had a history of regular menstrual cycles (no missed periods, cycle occurring every 26–28 days) and had not taken hormone-based medication in the 12 months prior to the study. The participant gave written informed consent and the study was approved by the University of California, Santa Barbara Human Subjects Committee and all experiments were performed in accordance with relevant guidelines and regulations.

### Study design

The participant underwent daily testing for 30 consecutive days, with the first test session determined independently of cycle stage for maximal blindness to hormone status. The participant began each test session with a behavioral assessment questionnaire followed by an immersive reality spatial navigation task (neither reported here, see^37^. Time-locked collection of blood draws and MRI experiments were followed each day (see^37^ for experimental timeline). Serum and whole blood started each day at 10:00 a.m. (± 30 min) when the participant gave a blood sample. Endocrine samples were collected, at minimum, after two hours of no food or drink consumption (excluding water). The participant refrained from consuming caffeinated beverages before each test session. MRI sessions followed venipuncture (± 30 min) and lasted one hour, consisting of structural and functional MRI sequences.

### Endocrine procedures

A licensed phlebotomist inserted a saline-lock intravenous line into either the dominant or non-dominant hand or forearm daily to evaluate hypothalamic-pituitary–gonadal axis hormones, including serum levels of gonadal hormones (17β-estradiol, progesterone and testosterone) and pituitary gonadotropins (luteinizing hormone (LH) and follicle stimulating hormone (FSH)). One 10 cc mL blood sample was collected in a vacutainer SST (BD Diagnostic Systems) each session. The sample clotted at room temperature for 45 min until centrifugation (2000×*g* for 10 min) and were then aliquoted into three 1 mL microtubes. Serum samples were stored at − 20 °C until assayed. Serum concentrations were determined via liquid chromatography-mass spectrometry (for all steroid hormones) and immunoassay (for all gonadotropins) at the Brigham and Women’s Hospital Research Assay Core. Assay sensitivities, dynamic range, and intra-assay coefficients of variation (respectively) were as follows: estradiol. 1 pg/mL, 1–500 pg/mL, < 5% relative standard deviation (*RSD*); progesterone, 0.05 ng/mL, 0.05–10 ng/mL, 9.33% *RSD*; testosterone, 1.0 ng/dL, < 4% *RSD.* FSH and LH levels were determined via chemiluminescent assay (Beckman Coulter). The assay sensitivity, dynamic range, and the intra-assay coefficient of variation were as follows: FSH, 0.2 mIU/mL, 0.2–200 mIU/mL, 3.1–4.3%; LH, 0.2 mIU/mL, 0.2–250 mIU/mL, 4.3–6.4%.

### MRI acquisition

The participant underwent a daily magnetic resonance imaging scan on a Siemens 3 T Prisma scanner equipped with a 64-channel phased-array head coil. High-resolution anatomical scans were collected using a *T*_*1*_-weighted magnetization prepared rapid gradient echo (MPRAGE) sequence (TR = 2500 ms, TE = 2.31 ms, TI = 934 ms, flip angle = 7°, 0.8 mm thickness) followed by a gradient echo fieldmap (TR = 758 ms, TE_1_ = 4.92 ms, TE_2_ = 7.38 ms, flip angle = 60°). Next, the participant completed a 10-min resting-state fMRI scan using a ${T}_{2}^{*}$-weighted multiband echo-planar imaging (EPI) sequence sensitive to the blood oxygenation level-dependent (BOLD) contrast (TR = 720 ms, TE = 37 ms, flip angle = 56°, multiband factor = 8; 72 oblique slices, voxel size = 2 mm^3^). To minimize motion, the head was secured with a custom fitted foam head case (days 8–30; https://caseforge.co/). Overall motion (mean framewise displacement; FWD) was negligible, with fewer than 130 microns of motion on average each day. Motion was not correlated with estradiol concentrations (Spearman’s $r = -.06, \; p = .758$) but was correlated with progesterone concentrations (Spearman’s *r* = .42, *p* = .020). However, extensive preprocessing steps were taken to minimize motion bias (see “fMRI preprocessing”).

### fMRI preprocessing

Preprocessing was performed using the Statistical Parametric Mapping 12 software (SPM12, WeLcome Trust Centre for Neuroimaging, London) in MATLAB. Functional data were realigned and unwarped to correct for head motion and geometric deformations, and the mean motion-corrected image was coregistered to the daily high-resolution anatomical image. All scans were then registered to a subject-specific anatomical template created using Advanced Normalization Tools’ (ANTs) multivariate template construction. A 4 mm full-width half-maximum (FWHM) isotropic Gaussian kernel was applied. Global signal scaling (median = 1000) was applied to account for transient fluctuations in signal intensity across space and time, and voxelwise time series were linearly detrended. Residual BOLD signal from each voxel was extracted after removing the effects of head motion and five physiological noise components (CSF + white matter signal). Additionally, mean signal from bilateral cerebral cortex within 7 mm of the cerebellum was included as a nuisance regressor to further isolate cerebellar signal^13^. Motion was modeled based on the Friston-24 approach, using a Volterra expansion of translational/rotational motion parameters, accounting for autoregressive and nonlinear effects of head motion on the BOLD signal^87^. All nuisance regressors were detrended to match the BOLD time series.

### Resting-state functional connectivity analysis

Functional network nodes were defined based on the Ren et al.^45^ 100-node cerebellar parcellation. Nodes were assigned to Buckner et al.^13^ seven-network cerebellar atlas based on spatial overlap using a consensus, ‘winner-take-all’ approach: for each parcel, voxels were assigned a network label based on the Buckner atlas; the whole parcel was then assigned to a network given the plurality of network labels across its voxels. This connectivity-based parcellation was selected because it is superior among existing cerebellar atlases with respect to accuracy of functional connectivity detection, node homogeneity, and individual identification^45^. Additionally, a 100-node atlas was preferred over other node atlas options (10 and 300 node) because it exhibited more moderate centering of each node and symmetry between the two hemispheres^45^.

Each day, a summary time course was extracted per node by taking the first eigenvariate across functional volumes^88^. The regional timeseries was then decomposed into frequency bands using a maximal overlap discrete wavelet transform (Daubechies extremal phase filter, length = 8). Low-frequency fluctuations in wavelets 3–6 (~ 0.01–0.17 Hz) were selected for subsequent connectivity analysis^89^. Finally, we estimated the *spectral* association between regional time series using magnitude-squared coherence: this yielded a 99 × 99 functional association matrix for each day, whose elements indicated the strength of functional connectivity between all pairs of nodes (FDR-threshold at $q < .05$). Note that although the Ren “100-node” parcellation was applied, only 99 nodes were available to be analyzed. The functional atlas deployed here was created using a normalized cut spectral clustering (N-cut) approach and one caveat of this approach is that the number of nodes may be less than the setting number (K;^90^).

### Statistical analysis

In order to relate cerebellar-hormone relationships to those observed in cerebral networks, we applied the statistical analyses reported on in Pritschet et al.^37^ (see for more detailed methods explanation). In short, day-by-day variation in functional connectivity associated with fluctuations in estradiol and progesterone was assessed through an edgewise regression analysis. Data were $Z$-transformed and edgewise coherence was regressed against the hormonal time series to capture day-by-day variation in connectivity relative to hormonal fluctuations. For each model, we computed robust empirical null distributions of test-statistics ($\beta /SE$) via 10,000 iterations of nonparametric permutation testing and we report only those edges surviving a conservative threshold of $p<.001$ to avoid over-interpretation of effects.

To determine the general direction of hormone-related associations with edgewise coherence, we took the thresholded statistical parametric maps for each model and estimated *nodal association strengths* per graph theory’s treatment of signed, weighted networks. That is, positive and negative association strengths were computed independently for each of the 99 nodes by summing the suprathreshold positive/negative edges linked to them. We then assessed mean association strengths (± 95% confidence intervals) in each direction across the various networks in our parcellation.

Here, the 99 nodes were grouped into networks based on their spatial association with large-scale cerebellar functional networks^13,45^. Through this approach, a total of six cerebral networks are represented in the cerebellum: FCN, DMN, VAN, DAN, SMN, and Limbic Network. The primary Visual Network is not represented in the cerebellum.

Next, we assessed associations between sex hormones and macroscale cerebellar network topologies. Briefly, we computed measures of *between-network* integration (the participation coefficient; i.e. the average extent to which network nodes are communicating with other networks over time) and *within-network* integration (global efficiency; quantifying the ostensible ease of information transfer across nodes inside a given network). To obtain these metrics for each day, the full (99 × 99) FDR-thresholded coherence matrices were subdivided into network matrices as defined by our parcellation. We then computed participation coefficients and global efficiencies for each network using the relevant functions for weighted graphs in the Brain Connectivity Toolbox^91^. Subsequently, a linear regression analysis was conducted. Linear models were initially fit across the complete dataset: an examination of residuals across each network/metric/hormone combination commonly revealed experiment day one as a potential outlier, with a median absolute deviation > 3 relative to the overall residual densities. We therefore removed it and re-ran the analysis: data were *Z*-scored, residualized on motion (mean FWD), and models were re-fit (*p*-values reported are FDR-corrected at a level of *q* < .05).

## Supplementary information

Supplementary Information.

## Data Availability

The dataset is openly available at https://openneuro.org/datasets/ds002674.

## Code availability

Code is available at https://github.com/tsantander/PritschetSantander2020_NI_Hormones.
