# Supplementary material for: Cerebellar network organization across the human menstrual cycle
Source: Sci Rep. 2020 Nov 26;10:20732. doi: 10.1038/s41598-020-77779-4 (PMC7691518; doi:10.1038/s41598-020-77779-4)
Supplement: Supplementary file 1 — Supplementary Information. [file 41598_2020_77779_MOESM1_ESM.docx]

**Supplementary Materials**

**Cerebellar network organization across the human menstrual cycle**

Morgan Fitzgerald, Laura Pritschet, Tyler Santander, Scott T. Grafton, & Emily G. Jacobs

| **Table S1.** Gonadal and pituitary hormones | | | |
| --- | --- | --- | --- |
|  | **Follicular** | **Ovulatory** | **Luteal** |
|  | Mean (SD) | Mean (SD) | Mean (SD) |
|  | *standard range* | *standard range* | *standard range* |
| Estradiol (pg/mL) | 37.9 (15.9) | 185.3 (59.0) | 85.4 (26.4) |
|  | *12.5-166.0* | *85.8-498.0* | *43.8-210* |
| Progesterone (ng/mL) | 0.2 (0.2) | 0.2 (0.2) | 9.5 (4.8) |
|  | *0.1-0.9* | *0.1-120* | *1.8-23.9* |
| LH (mIU/mL) | 5.9 (0.7) | 21.7 (16.4) | 5.5 (2.0) |
|  | *2.4-12.6* | *14.0-95.6* | *1.0-11.4* |
| FSH (mIU/mL) | 6.5 (1.2) | 8.1 (3.6) | 4.8 (1.3) |
|  | *3.5-12.5* | *4.7-21.5* | *1.7-7.7* |

*Note.* Standard reference ranges based on aggregate data from LabCorp (<https://www.labcorp.com/>).

| **Table S2.** Linear regression models for global efficiency v. hormones | | | | |
| --- | --- | --- | --- | --- |
| **Outcome** | **Network** | **Estimate** | **SE** | **T*(p)*** |
| Estradiol | Limbic | 0.31 | 0.18 | 1.69(.103) |
|  |  | F(1,28) = 2.84, *p* = .103; *R^2^_Adj_* = 0.06 | | |
|  | Frontal Control | -0.33 | 0.18 | -1.85(.076) |
|  |  | F(1,28) = 3.41, *p* = .076; *R^2^_Adj_* = 0.08 | | |
|  | Default Mode | -0.14 | 0.19 | -0.75(.462) |
|  |  | F(1,28) = 0.56, *p* = .462; *R^2^_Adj_* = -0.02 | | |
| Progesterone | SomatoMotor | -0.32 | 0.18 | -1.78(.086) |
|  |  | F(1,28) = 3.17, *p* = .086; *R^2^_Adj_* = 0.07 | | |
|  | Limbic | 0.36 | 0.18 | 2.01(.054) |
|  |  | F(1,28) = 4.05, *p* = .054; *R^2^_Adj_*= 0.10 | | |
|  | Frontal Control | -0.15 | 0.19 | -0.77(.451) |
|  |  | F(1,28) = 0.59, *p* = .451; *R^2^_Adj_* = -0.02 | | |
|  | Default Mode | 0.22 | 0.19 | 1.16(.258) |
|  |  | F(1,28) = 1.33, *p* = .258; *R^2^_Adj_* = 0.01 | | |

| **Table S3.** Linear regression models for participation v. hormones | | | | | | |
| --- | --- | --- | --- | --- | --- | --- |
| **Outcome** | **Network** | **Estimate** | **SE** | | | **T*(p)*** |
| Estradiol | SomatoMotor | 0.19 | 0.19 | | | 0.99(.329) |
|  |  | F(1,28) = 0.99, *p* = .329; *R^2^_Adj_* = 4.10 x 10^-4^ | | | | |
|  | Ventral Attention | -0.34 | 0.18 | | | -1.87(.072) |
|  |  | F(1,28) = 3.51, *p* = .072; *R^2^_Adj_* = 0.08 | | | | |
|  | Dorsal Attention | -0.19 | 0.19 | | | -1.03(.314) |
|  |  | F(1,28) = 1.05, *p* = .314; *R^2^_Adj_* = 0.002 | | | | |
|  | Limbic | 0.03 | 0.19 | | | 0.16(.872) |
|  |  | F(1,28) = 0.03, *p* = .872; *R^2^_Adj_* = -0.04 | | | | |
|  | Frontal Control | -0.32 | 0.18 | | | -1.78(.086) |
|  |  | F(1,28) = 3.17, *p* = .086; *R^2^_Adj_* = 0.07 | | | | |
|  | Default Mode | -0.27 | 0.19 | | | -1.48(.149) |
|  |  | F(1,28) = 2.20, *p* = .149; *R^2^_Adj_* = 0.04 | | | | |
| Progesterone | SomatoMotor | 0.34 | 0.18 | | | 1.88(.072) |
|  |  | F(1,28) = 3.51, *p* = .072; *R^2^_Adj_* = 0.08 | | | | |
|  | Ventral Attention | -0.16 | 0.19 | | | -0.86(.398) |
|  |  | F(1,28) = 0.74, *p* = .398; *R^2^_Adj_*= -0.01 | | | | |
|  | Dorsal Attention | -0.12 | 0.19 | | | -0.62(.539) |
|  |  | F(1,28) = 0.39, *p* = .539; *R^2^_Adj_* = -0.02 | | | | |
|  | Limbic | 0.07 | 0.19 | | | 0.36(.722) |
|  |  | F(1,28) = 0.13, *p* = .722; *R^2^_Adj_* = -0.03 | | | | |
|  | Default Mode | -0.17 | | .19 | -0.92(.366) | |
|  |  | F(1,28) = 0.84, *p* = .366*; R^2^_Adj_* = -0.01 | | | | |


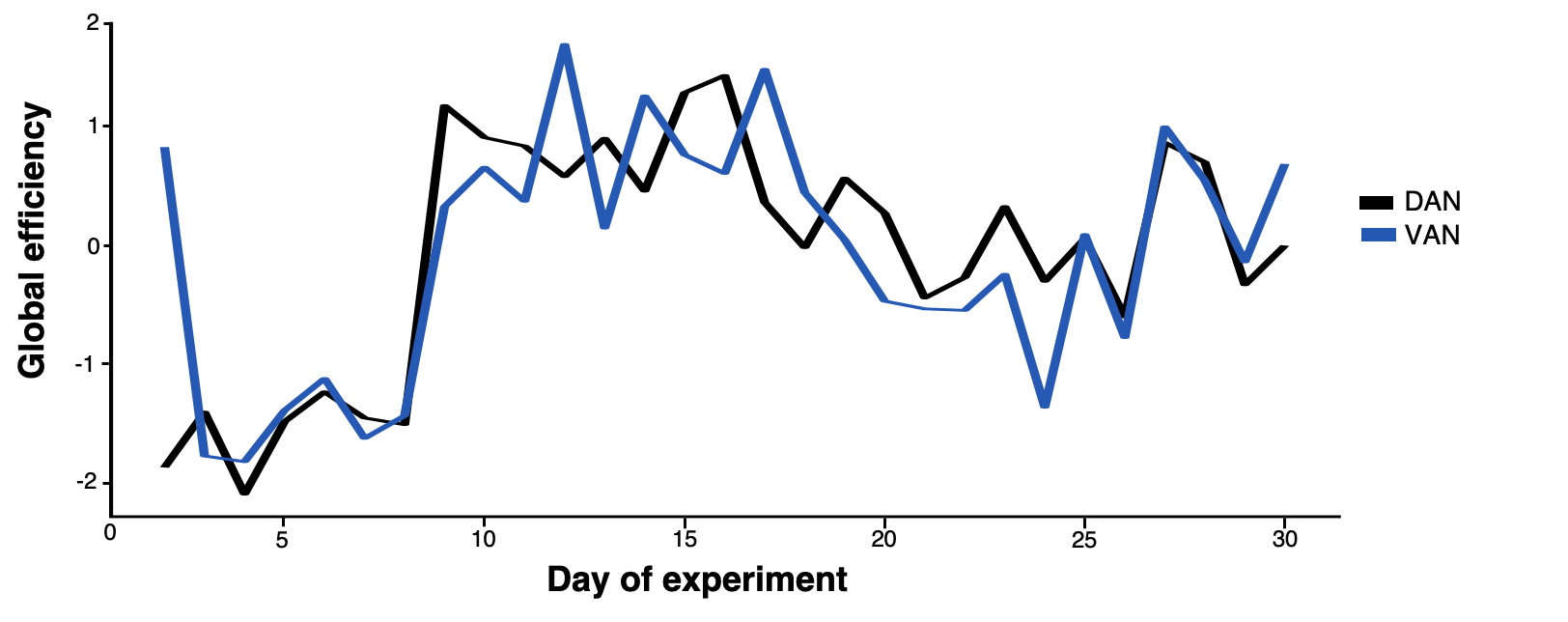


**Figure S1. Day-by-day variation in network topology across the 30-day experiment.** Standardized global efficiency, a measure of within-network integration, reflects the ostensible ease of information transfer across clusters inside a given network. Abbreviations: DAN, Dorsal Attention Network; VAN, Ventral Attention Network.

**Figure S2. Sex hormones display network-specific associations with whole-cerebellar coherence.** Day-by-day associations between progesterone (left) and estradiol (right) with coherence grouped by network (black squares). Lighter colors indicate increased coherence with higher concentrations of sex hormones; dark colors indicate the reverse. Results are empirically-thresholded via 10,000 iterations of nonparametric permutation testing (*p <* .001*).* Abbreviations: DMN, Default Mode Network; DAN, Dorsal Attention Network; FCN, Frontal Control Network; SMN, SomatoMotor Network; VAN, Ventral Attention Network.


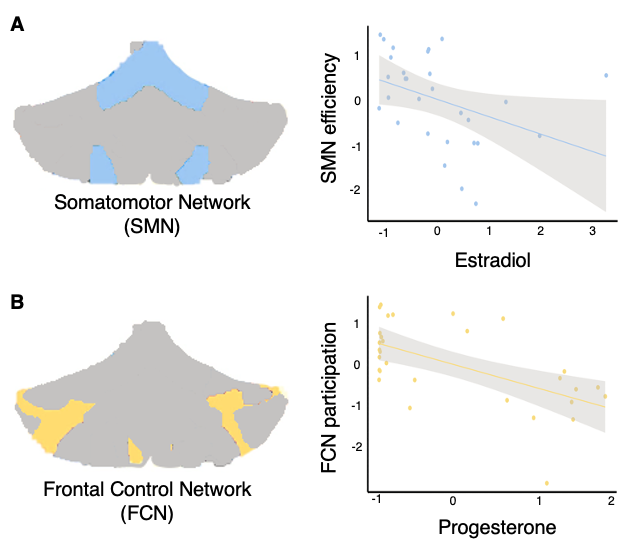


**Figure S3. Graph theory metrics reveal relationships between sex hormones and intra- and inter-network integration. (A)** Illustration (left) depicts nodes belonging to the SomatoMotor Network (SMN). Global efficiency, a measure of within-network integration, was calculated to reflect the ostensible ease of information transfer across clusters inside a given network and was regressed against sex hormone concentrations. Here, scatter plots (right) depict significant associations (*p* < .05) between estradiol and SMN efficiency.  **(B)** Illustration (left) depicts nodes belonging to the Frontal Control Network (FCN). Participation coefficient, which represents a measure of between-network integration defined as the average extent to which network clusters are communicating with other networks over time, was regressed against sex hormone concentrations. Here, the scatter plot (right) depicts significant associations (*p* < .05) between progesterone and FCN participation.


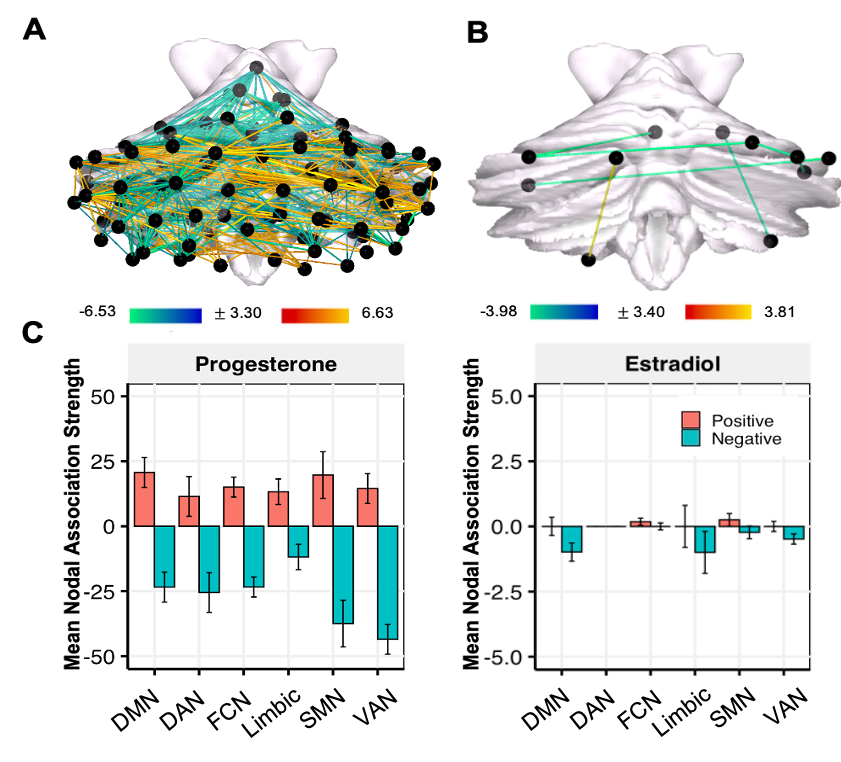


**Figure S4. Use of non-smoothed data had minimal impact on brain-hormone associations**. **(A**) Day-by-day associations between progesterone and coherence using non-smoothed data. Hotter colors indicate increased coherence with higher concentrations of estradiol; cool colors indicate the reverse. Results are empirically-thresholded via 10,000 iterations of nonparametric permutation testing (*p <* .001*).* Nodes without significant edges are omitted for clarity. **(B)** Day-by-day associations between estradiol and coherence for non-smoothed data. **(C)** Mean nodal association strengths by network and hormone for non-smoothed data. Error bars give 95% confidence intervals. ‘Positive’ refers to the average magnitude of positive associations (e.g. stronger coherence with higher estradiol). Abbreviations: DMN, Default Mode Network; DAN, Dorsal Attention Network; FCN, Frontal Control Network; SMN, SomatoMotor Network; VAN, Ventral Attention Network. Statistical maps of edgewise coherence v. hormones were visualized using the Surf Ice software (https://www.nitrc.org/projects/surfice/).

**Figure S5. Filtered head motion estimates across the 30-day experiment.** Motion was further estimated using a low-pass filtering approach aimed to reduce high-frequency contamination. Mean framewise displacement did not exceed 21 microns, confirming consistently low motion throughout the experiment. Motion is plotted both to reflect the typical range observed in brain imaging studies (left) and zoomed in (right). Motion on days 1-8 was limited with ample head and neck padding; motion on days 9-30 was limited using a molded headcase custom fit to the participant’s head.
